# In-hospital complications following primary total hip and knee arthroplasty in octogenarian and nonagenarian patients

**DOI:** 10.1007/s10195-013-0262-y

**Published:** 2013-08-29

**Authors:** Shashi K. Nanjayan, Girish N. Swamy, Sunil Yellu, Sachin Yallappa, Tarek Abuzakuk, Robert Straw

**Affiliations:** Royal Derby Hospital, Uttoxeter New Road, Derby, DE22 3NE UK

**Keywords:** In-hospital complications, Octogenarian, Nonagenarian, Arthroplasty

## Abstract

**Background:**

As life expectancy of patients increases, more elderly patients are undergoing primary total hip arthroplasty (THA) and total knee arthroplasty (TKA). There is a general perception of increased risk of complications in elderly patients. Our objective was to analyse the incidence of in-hospital medical and surgical complications following THA and TKA in octogenarian and nonagenarians.

**Materials and methods:**

This was a prospective review of 202 consecutive patients aged more than 80 years who underwent total hip and total knee arthroplasty (101 THA, 101 TKA) over an 18-month period. In this single-centre observational study, collected data included patient demographics, American Society of Anethesiologists (ASA) grade, length of hospital stay and peri-operative medical and surgical complications during their hospital stay.

**Results:**

Median age of patients was 83 years. Median ASA grade was 3. Mean length of hospital stay was 7.5 days. There were 14 major systemic complications in the THA group and 13 in the TKA group. While 1 major local complication occurred in each group, there were 6 minor local complications in THA and 7 in the TKA group. All the complications occurred within 5 post-operative days. There was no in-hospital mortality.

**Conclusion:**

In our study, we found that the incidence of peri-operative medical and surgical complications is higher in those over 80 years, compared to the published literature in patients of all age groups undergoing THA and TKA. Awareness of a higher incidence of major systemic complications should alert the treating surgeon to carry out comprehensive peri-operative management in this subset of patients, which could lead to better outcomes.

## Introduction

Primary total knee arthroplasty (TKA) and primary total hip arthroplasty (THA) are the most commonly performed elective orthopaedic operations in the United Kingdom. The average age of patients undergoing TKA is 67.4 years, and those undergoing THA is 67.2 years [1]. The life expectancy at birth for the UK population was 78.2 years for males and 82.3 years for females between 2008–2010 [2].

As the life expectancy of patients is increasing, more elderly patients are undergoing THA and TKA due to increasing incidence of osteoarthritis in this age group. The recent advances in health care have enabled these patients to undergo major procedures like TKA and THA, helping them to pursue more active lifestyles. There is a general perception of increased risk of ‘in-hospital’ complications in elderly patients undergoing TKA or THA. This could be due to their general health status and their associated medical co-morbidities. Advancing age is reported to be a significant risk factor in the incidence of in-hospital complications [3].

The objective of this study was to analyse the incidence of in-hospital peri-operative medical and surgical complications following THA and TKA in patients with advanced age, that is, in octogenarians and nonagenarians, and to compare the same with the published literature.

## Materials and methods

A prospective review of 202 consecutive patients aged more than 80 years, who underwent total hip and total knee arthroplasty (101 THA, 101 TKA) during an 18-month period was carried out.

The inclusion criterion was:

1. All patients older than 80 years of age undergoing unilateral primary THA or primary TKA

Exclusion criteria were:

1. Patients undergoing revision THA or revision TKA
2. Patients undergoing unicompartmental TKA
3. Patients under the age of 80 years undergoing primary THA and primary TKA

No enrolled patients were excluded during the study.

All the patients gave informed consent prior to being included in the study. All the operations were unilateral and primary arthroplasties. In this single-centre observational study, collected data included patient demographics, American Society of Anethesiologists (ASA) grade, length of hospital stay and peri-operative medical and surgical complications during their hospital stay. All these patients underwent pre-operative assessment, a few weeks prior to the surgery, led by the surgeon and the anaesthetist. If any medical issues were identified, they were optimised prior to the surgery. Unless contraindicated or an attempt failed, all the patients received spinal anaesthesia with an additional regional block. All operations were performed in dedicated orthopaedic operating theatres, which have a laminar air flow system. Trainees performed 19.3 % (*n* = 39) of the operations under the supervision of consultants, while the consultant surgeons performed 80.7 % (*n* = 163) of the operations.

Forty-one of the THAs were performed using a posterior approach, and 60 with a modified anterolateral approach. Different surgical approaches for THA were used according to the surgeon’s preference. Ninety-nine were cemented THA and 2 were hybrid THA.

All TKAs were performed with the use of a tourniquet and medial parapatellar approach. All TKA implants were metal backed and fixation was cemented. We did not use any wound drains for the total hip arthroplasty. Eighty-seven patients had drains for TKA, which were removed routinely at 24 h. Placement of drains following TKA was according to the surgeon’s preference.

A multimodal method was used for venous thromboembolism (VTE) prophylaxis, using TED stockings, foot pumps and aspirin or low molecular weight heparin (enoxaparin 40 mg subcutaneously, once daily) for 6 weeks post-operatively. Intravenous antibiotics were given at induction of anaesthesia, with two further doses in the next 24 h. All patients were encouraged to mobilise the same day or the day after surgery.

Check radiographs, full blood count, urea and electrolyte were tested the following day, unless the clinical condition of the patient dictated earlier investigation.

All the complications that occurred during the patients’ stay in hospital were recorded in the database.

We analysed the age, gender, height, weight, BMI, type of anaesthesia, ASA grade, surgeon’s grade and type of thromboprophylaxis and drop in post-operative haemoglobin as risk factors for occurrence of complications.

The complications were classified as major and minor systemic complications and major and minor local complications as used in studies by Parvizi et al. [3] and Pulido et al. [4].

Statistical analysis was done using R Software. Univariate regression analysis was performed on all risk factors for complications. Multiple logistic regression analysis was performed on all variables that were significant in univariate analysis. For the univariate analysis, a *p* value of <0.1 was considered significant. For all other tests, a *p* value of <0.05 was considered significant.

## Results

The median age of patients was 83 years (IQR 80–95 years). There were 150 patients aged between 80 and 85 years, 39 patients between 86 and 90 years and 13 aged more than 90 years. There were 126 female patients and 76 male.

The most common ASA grade was either ASA 2 (*n* = 111) or ASA 3 (*n* = 85). The median BMI was 28 (IQR 25–31). Median height was 162 cm (IQR 156–170 cm).

Median weight was 75 kg (IQR 65–86 kg). Mean length of hospital stay was 7.5 days. Nearly 24 % (*n* = 48) of patients required blood transfusion.

We found 14 major systemic complications in the THA group and 13 major systemic complications in the TKA group. There were 16 minor systemic complications in THA and 17 minor systemic complications in TKA. One major local complication was found in both THA and TKA groups (Table 1). All complications occurred within 5 post-operative days.

**Table 1:** Incidence of complications

Major systemic complications	THA (*n* = 101)	TKA (*n* = 101)
Cardiovascular		
Pulmonary embolism	0	2
Atrial fibrillation	3	4
Myocardial infarction	1	1
Pulmonary oedema	1	0
Arrhythmia		
Deep vein thrombosis	0	0
Neurological		
Stroke	0	1
Pulmonary		
Respiratory arrest (opioid induced)	1	0
Pneumonia	4	3
Gastrointestinal		
Gastrointestinal bleed	2	0
Altered liver function	1	0
Renal/fluid and electrolyte imbalance		
Acute kidney injury	1	1

Minor systemic complications		
Cardiovascular		
Angina	1	0
Sinus tachycardia	1	1
Hypotension	4	1
Neurological		
Confusion/delirium	4	5
Urinary		
Urinary tract infection	4	1
Urinary retention	1	1
Fluid and electrolyte imbalance		
Hypokalemia	1	3
Hyponatremia	1	3
Gastrointestinal		
Diarrhoea (*Clostridium difficile*–negative)	3	2
General		
Fall in the hospital	1	1
Vasovagal episode/unresponsiveness	1	1
In-hospital mortality	0	0

Local complications	THA (*n* = 101)	TKA (*n* = 101)
Major		
Dislocation	1	0
Peripheral nerve injury (foot drop)	0	1
Vascular injuries	0	0

Minor		
Skin tear	1	0
Wound haematoma	1	1
Wound leakage	1	2
Superficial wound infection	1	1
Blisters	1	2
Cellulitis	1	2

We did not have any in-hospital mortality in our cohort of patients.

Univariate regression analysis revealed that significant factors for complications were ‘length of stay in the hospital’ and reception of ‘blood transfusion’ (Table 2).

**Table 2:** Univariate analysis of variables

Variable	Statistic value	*p*′ value
TKA vs THA	0.19	0.66
Age	1.90	0.31
Gender	2.20	0.14
Height	1.21 (*z* value)	0.23
Weight	0.13 (*z* value)	0.90
BMI	0.89 (*z* value)	0.37
Surgeon grade (trainee vs consultant)	0.10	0.75
Type of anaesthesia	3.23	0.52
Type of DVT prophylaxis	2.97	0.81
ASA grade	1.29	0.73
Length of hospital stay	5.53	<0.00001
Transfusion	17.32	<0.012
Drop in haemoglobin (<4 vs >4 mg/dl)	4.02	0.13

Multivariate logistic regression using the Akaike information criterion (AIC) revealed that patients who stayed in the hospital for more than 5 days (*p* < 0.00001) and those who received blood transfusion had a significant *p* value (*p* < 0.012). Likelihood of suffering a complication was slightly higher in these groups (LOS — odds ratio 3.12 and transfusion — odds ratio 3.38). This probably reflects the fact that a patient who developed a complication probably stayed a few days more in the hospital and was more likely to receive a blood transfusion.

## Discussion

With increasing advances and standards in the peri-operative care of patients, in-hospital complications are expected to be low. But, in older patients, especially octogenarians and nonagenarians, there is an increased incidence of complications [4–7].

Clement et al. [5] in their prospective study comparing a selected group of patients aged more than or equal to 80 years and a control group aged between 65 and 74 years, reported increased medical complications in patients older than 80 years. The older group had a longer hospital stay for both THA and TKA.

In a larger series of 15,383 patients undergoing total joint arthroplasties, Pulido et al. [4] reported a major systemic complication in 2.02 % of primary THA and 4.91 % of primary TKA, as well as minor systemic complications in 3.17 % of primary THA and 3.40 % of primary TKA. We found a much higher incidence of major complications (14 % in the THA group and 13 % in the TKA group). The incidences of minor systemic complications were also found to be higher in our group (16 % in THA and 17 % in TKA).

They reported the incidence of major local complications at 0.48 % for primary THA and 0.83 % for primary TKA, whereas we found 1 % incidence in our cohort. Minor local complications were also increased in our cohort; 6 % in THA and 7 % in TKA when compared to 1.95–2.54 % in their series. Incidences of these complications are more in our group. Our cohort was of older patients with a median age of 83 years, whereas the mean age in the Pulido et al. [4] series was 61 years in males and 65 years in females.

Wurtz et al. [8] reported 27 % (*n* = 11) medical-related complications and 15 % (*n* = 6) hip-related complications in the octogenarian population undergoing THA. We found a similar major systemic complication rate in our cohort.

The most common complication observed in our study was an episode of post-operative cognitive dysfunction (POCD). There were 9 cases of POCD in our study (4 in THA and 5 in TKA). Investigation carried out in these patients revealed urinary tract infection in one patient and hyponatremia in another patient, which resolved after instituting the appropriate treatment. In the remaining 7 patients, no identifiable cause was found, and it resolved prior to discharge. Confusion in hospitalised elderly patients is multifactorial and its incidence increases with increasing age [9]. Older age, peri-operative use of narcotics, polypharmacy, acute pain, urinary catheter, addition of 3 or more medications in 24–48 h, anaemia, fluid and electrolyte imbalance, greater surgical blood loss and intra-operative transfusion have been reported to be triggers of post-operative cognitive dysfunction [10].

The incidence of POCD in elderly patients has been reported to be between 5 and 15 % [11], and increases to about 25 % in older patients in the immediate post-operative days [12]. The decreasing trend in the incidence of POCD is probably because of the increasing standards of peri-operative care.

Larger series have reported clear age- and sex-specific frequencies of myocardial infarction (MI) in the peri-operative period following THA and TKA. The increases in incidence of adverse events like MI, pulmonary embolism (PE), DVT and death was slightly more in patients aged more than 80 when compared to younger patients [13, 14]. Two cases of PE in our patients were, after TKA, proven on a computed tomographic pulmonary angiogram (CTPA). One case of MI in each THA and TKA were treated according to the hospital cardiology guidelines.

One study has shown that incidence of DVT did not differ significantly with age, sex or type of operation [13]. There was no incidence of DVT in our patients, although 7 patients (5 TKA, 2 THA) underwent DVT study (vascular ultrasound) with suspected DVT, but none of them were proven positive.

Atrial fibrillation (AF) was observed in 3 % of THA and 4 % of TKA in our series. A larger series [4], with a mean age between 61 years for males and 65 years for females reported an incidence of 0.4 % in THA and 0.5 % in TKA. Increasing age is a known, independent variable in the incidence of AF [15]. The findings in our series are similar.

ASA grade as a comorbidity index is validated in hip fracture surgery [16] and it is a useful indicator of peri-operative patient health status [17]. We found that the ASA grade was not a key predictor of occurrence of complication in this cohort of patients.

Patel et al. [18] showed that morbid obesity was an independent risk factor for prolonged wound drainage following joint arthroplasties, irrespective of type of VTE prophylaxis. We had two cases of wound haematoma (1 THA, 1 TKA) and 3 cases of wound leakage (1 THA, 2 TKA). The mean BMI of our patients was 28 and our usual DVT prophylaxis was multimodal (aspirin 150 mg + TED stockings + peri-operative foot pump). Neither BMI nor type of DVT prophylaxis was a significant variable for occurrence of in-hospital complications in our study.

Acute vascular complications are very rare following THA and TKA [19]. Although risk of arterial calcification is slightly higher in older patients, rendering them vulnerable to arterial injuries, fortunately, we did not have any such complications.

In-hospital mortality has been shown increase at least ninefold in patients older than 85 years, when compared to patients aged between 45 and 64 years [20]. ASA grade is a significantly related to post-operative death [17]. We did not have any mortality in our cohort despite the mean age of 83 years. Nevertheless, this cannot undermine the importance of careful and meticulous planning of peri-operative care needed for octogenarians, as THA and TKA is increasingly performed in this population.

Incidence of these complications was slightly more in our group when compared to the largest series published in the literature [4], but our cohort was of older patients with a median age of 83 years, whereas the mean age in the Pulido et al. [4] series was 61 years in males and 65 years in females.

A limitation of our series was that our cohort was relatively small, keeping in mind the frequency of THA and TKA.

The option of recording ‘in-hospital complication’ in the National Joint Registers should be considered. This would allow better capture of the data on a national database, which would then allow better understanding and thus better standards of care to our patients. The analysis of more data can be used to obtain better informed consent from this cohort of patients.

In conclusion, our study found that the incidence of peri-operative medical and surgical complications is more common in those over 80 years old compared to the published literature in patients of all age groups undergoing total hip and knee arthroplasty. Awareness of the higher incidence of major systemic complications in this subset of patients should alert the treating surgeon to carry out a comprehensive pre-operative evaluation to optimise the co-existing medical condition. Further, vigilant post-operative management of these patients could lead to better outcomes.
